# Microbes in the coral holobiont: partners through evolution, development, and ecological interactions

**DOI:** 10.3389/fcimb.2014.00176

**Published:** 2015-01-07

**Authors:** Janelle R. Thompson, Hanny E. Rivera, Collin J. Closek, Mónica Medina

**Affiliations:** ^1^Civil and Environmental Engineering Department, Massachusetts Institute of TechnologyCambridge, MA, USA; ^2^Department of Biology, Woods Hole Oceanographic InstitutionWoods Hole, MA, USA; ^3^Department of Biology, Pennsylvania State UniversityUniversity Park, PA, USA

**Keywords:** coral, holobiont, metamorphosis, biological, symbiosis, pollution and global change, ecosystem, bacterial interactions

## Abstract

In the last two decades, genetic and genomic studies have revealed the astonishing diversity and ubiquity of microorganisms. Emergence and expansion of the human microbiome project has reshaped our thinking about how microbes control host health—not only as pathogens, but also as symbionts. In coral reef environments, scientists have begun to examine the role that microorganisms play in coral life history. Herein, we review the current literature on coral-microbe interactions within the context of their role in evolution, development, and ecology. We ask the following questions, first posed by McFall-Ngai et al. (2013) in their review of animal evolution, with specific attention to how coral-microbial interactions may be affected under future environmental conditions: (1) How do corals and their microbiome affect each other's genomes? (2) How does coral development depend on microbial partners? (3) How is homeostasis maintained between corals and their microbial symbionts? (4) How can ecological approaches deepen our understanding of the multiple levels of coral-microbial interactions? Elucidating the role that microorganisms play in the structure and function of the holobiont is essential for understanding how corals maintain homeostasis and acclimate to changing environmental conditions.

## Introduction

Multicellular hosts harbor communities of beneficial microbes (Bosch and McFall-Ngai, 2011). Compelling evidence illustrates how microorganisms have facilitated the origin and evolution of animals and are integral parts of all animal life (McFall-Ngai et al., 2013). The coral holobiont is comprised of the coral animal and its associated microorganisms consisting of bacteria, archaea, fungi, viruses, and protists including the dinoflagellate algae *Symbiodinium* (Rohwer et al., 2002). The coral holobiont is a dynamic system, whose members fluctuate depending on environmental conditions and daily requirements (Shashar et al., 1993; Tanner, 1996; Ainsworth et al., 2011). In their review of animal evolution, McFall-Ngai et al. (2013) proposed that our understanding of microbes' role in the evolution of animal partners could be increased by examining mutual interactions and reciprocal influences during development and genomic evolution. In this review, we follow their framework to evaluate the role coral-microbe associations have played in (1) genome evolution in both host and microbial partners, (2) shaping and driving coral development, (3) modulating holobiont homeostasis, and (4) defining the ecology of interactions in the coral holobiont. We aim to review the current literature on coral-microbe interactions in order to create a consolidated resource of knowledge in an emerging and quickly developing field. As the symbiosis between corals and *Symbiodinium* represents a long-standing field of investigation (Trench, 1983, 1993; Weber and Medina, 2012) we will focus in this review on our emerging understanding of other microbial components of the holobiont that we collective refer to as the microbiota.

## How have corals and microorganisms affected each other's genomes?

Coral microbiome studies have revealed vast bacterial diversity co-existing with different host species (Rohwer et al., 2001; Dinsdale et al., 2008; Sunagawa et al., 2010; Morrow et al., 2012). Microbial associations over evolutionary time scales are likely to contribute to genome differentiation in both the host and its associated microbial partners, a process coined as hologenome evolution (Rosenberg et al., 2007). In light of evidence from multiple systems, we will discuss the different scenarios in which host-microbial interactions shape each other's genomes. These include co-evolutionary patterns, metabolic complementation, genomic modification through either genome reduction/expansion, or genetic exchange through horizontal gene transfer (HGT).

### Bacterial genome evolution in the holobiont

Bacterial genome size reflects evolutionary dependency of microbial species engaged in obligate symbiosis with multicellular hosts (Moran et al., 2008). Obligate symbionts exhibit the smallest genome sizes when compared to facultative endosymbionts or free-living bacteria (McCutcheon and Moran, 2012). Data on genome size reduction come from studies on insect endosymbiosis, but similar trends are likely to be observed in other tightly coupled host-microbe associations (Gerardo, 2013). While much of the evolutionary history between corals and their bacterial symbionts is yet to be elucidated, patterns of transmission are likely playing a role in host and symbiont genome evolution. If a bacterial symbiont is both vertically transmitted and an endosymbiont, its genome will be more likely to be reduced in size relative to other bacterial mutualists that have free-living stages. The coral-associated bacterial genomes that have been sequenced to date are from cultured facultative coral associates with a presumed free-living state and contain genomes that are of a similar size as their non-coral associated relatives (Reshef et al., 2008; Mavromatis et al., 2010; Santos et al., 2011; Bondarev et al., 2013; Neave et al., 2014). For example, the recently described Verrucomicrobia species *Coraliomargarita akajimensis* (3.7 Mb genome) was isolated from water adjacent to the coral *Galaxea fascicularis* (Mavromatis et al., 2010) is within the size range estimated for other recently sequenced Verrucomicrobia genomes from aquatic habitats (Martinez-Garcia et al., 2012). Other coral-associated genomes from Proteobacteria exceed 5 Mb which is typical for free-living Proteobacteria. These include an alpha-Proteobacterium *Pseudovibrio* strain FO-BEG1 (5.9 Mb genome) isolated from an enrichment culture of a black band diseased coral (Bondarev et al., 2013), the gamma-Proteobacteria *Endozoicomonas montipora* LMG24815 (5.6 Mb genome) isolated from *Montipora aequituberculata* (Yang et al., 2010) and *Vibrio coralliilyticus* a coral pathogen with a 5.5 Mb genome (Santos et al., 2011; Kimes et al., 2012).

Thanks to major developments in sequencing technology, we are no longer limited to studying the small fraction of coral-associated microbes that can be cultured. Indeed, if corals harbor co-evolved microbial symbionts these may not be readily cultured due to fastidious nutritional requirements and metabolic complementation. Culture-independent approaches such as sequencing of 16S rRNA genes has revealed high proportions of novel diversity associated with corals (Frias-Lopez et al., 2002; Rohwer et al., 2002; de Castro et al., 2010; Sunagawa et al., 2010; Fernando et al., 2014) and the dynamic genomic diversity of the microbiota has been revealed through metagenomics revealing shifts in the proportions of microbial genes for functions such as nutrient cycling and virulence following environmental perturbations (Yokouchi et al., 2006; Dinsdale et al., 2008; Vega Thurber et al., 2009). Despite these advances, identifying specific microbial activities in the holobiont and linking these to individual microbial populations that may share a co-evolutionary history with corals remains a challenge. Genome sequencing of single cells and expanded novel-cultivation-based approaches for isolation-based studies are potential tools to characterize currently uncultured microbial symbionts. Such efforts will improve the taxonomy of coral-associated microbes and will enable us to test hypothesis of coevolution and co-diversification in the coral holobiont.

### Emerging evidence for metabolic complementation

In addition to the well-established mutualistic nutritional benefits between corals and *Symbiodinium* (Muscatine and Porter, 1977; Muscatine, 1990; Shoguchi et al., 2013), other reciprocal relationships of metabolic-complementation could extend beyond the coral-*Symbiodinium* spectrum for example, to include metabolic interactions between bacteria and *Symbiodinium* or bacteria and the coral host. Metabolic complementation, where each partner produces gene products necessary for survival of the other, may be reflected in the genome composition of the partners (Gerardo, 2013). In a recent analysis of a tri-partite insect endosymbiosis (i.e., two bacterial endosymbionts and the host), extreme genome reduction was correlated with shared metabolic pathways across three taxa (Husnik et al., 2013). The time of symbiont acquisition however played a key role in driving gene loss in the different bacterial lineages.

The mainly oligotrophic habitat of corals, makes associations driven by metabolic complementation desirable, for instance association between diazotrophic bacteria and *M. cavernosa* has been shown to improve nitrogen fixation (Lesser et al., 2004). Corals are open systems, in which both obligate endosymbionts and other microbes may be closely interacting with the animal host in a temporally dynamic fashion, increasing the chance for such metabolic cooperation to develop. Indeed, the recently sequenced genome of the coral *Acropora digitifera* lacked a key enzyme that synthesizes the essential amino acid cysteine from homocysteine or serine (Shinzato et al., 2011; Dunlap et al., 2013). Cysteine thione β synthase (CBS) is missing also from two other *Acropora* species but it has been reported in multiple corals (Shinzato et al., 2011), suggesting metabolic complementation by microbial symbionts within *Acropora* (e.g., algal or other).

Evidence that metabolic interaction between holobiont members shaped genome evolution may take the form of genetic systems acquired by the host genome by HGT. Predicted genes for bacterial metabolic pathways i.e., the shikimic acid pathway for biosynthesis of aromatic amino acids, and the glyoxylate pathway for biosynthesis of carbohydrates, are present in the genome of the sea anemone *Nematostella* due to proposed HGT (Starcevic et al., 2008; Jackson et al., 2011). Similar evidence for potential HGT of the bacterial shikimic acid pathway into the coral *A. digitifera* has been presented (Shinzato et al., 2011; Shoguchi et al., 2013) pointing to the possible acquisition of the pathway by a common ancestor of the Anthozoa.

The role of HGT is largely unexplored within coral holobiont but new whole genome data from multiple members associated with a single coral host will reveal how prevalent HGT is in coral reef symbioses. Indeed studies from the human gut microbiome have shown that HGT is rampant among gut microbial associates where conditions may parallel those associated with the coral polyp (e.g., high bacterial densities and selective pressures from host) (Smillie et al., 2011). We will soon see an increase in whole genome data from multiple coral hosts, algal symbionts, as well as microbial genomes from these hosts, which will open our horizons to understand the extent of metabolic complementation and rate of HGT across holobiont members.

## How do microorganisms influence coral development?

**Figure 1:**
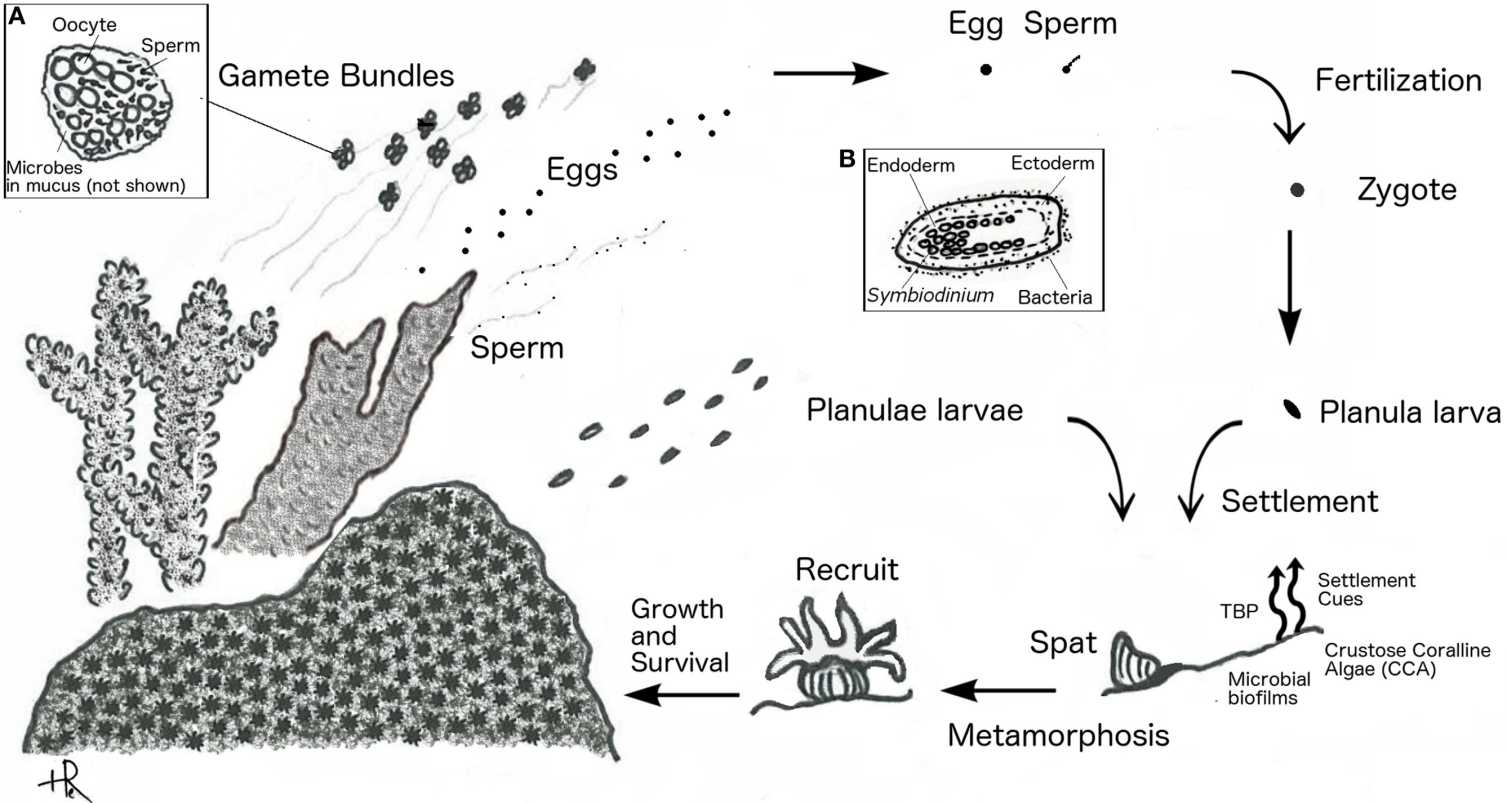
**Coral reproductive cycle**. Spawning corals (shown as branching) release gamete bundles, eggs, or sperm, and brooding corals (shown as massive) release planulae. Inset **(A)** shows close up of gamete bundle. Inset **(B)** shows close up of planula larva.

Microbial interactions can play a role at each of the stages in a coral's life cycle and even be crucial in settlement and metamorphosis. Scleractinian corals reproduce asexually through budding and fragmentation, and sexually by gamete spawning or larval brooding. Spawning corals release large quantities of eggs and sperm—either directly or packaged in gamete bundles—into the water column where fertilization and development occurs. Brooding corals, in contrast, release sperm but retain unfertilized eggs inside the gastrovascular cavity of their polyps. The sperm fertilize brooded eggs internally, where they develop before being released as planula larvae. A successful larval life cycle concludes with substrate selection, settlement, and metamorphosis to a polyp (Harrison, 2011). We examine how coral-microbe interactions facilitate the coral life cycle. We define the following transitions: (1) fertilization and spawning – fertilization of gametes and formation of a motile pelagic planula larva (2) settlement and metamorphosis – the selection of appropriate settlement substrate by the free-swimming planula and metamorphosis into polyp (3) coral colony formation – the asexual division of a single polyp into a juvenile colony (4) gamete formation and spawning – sexually mature colonies that produce and release gametes or brood larvae (Figure 1).

### Gametogenesis and spawning

Gamete formation in corals is not currently known to involve microbial partners, however emerging evidence of vertical and horizontal transmission supports a role for beneficial bacterial populations that associate with coral spawn. The transmission of symbionts in corals has been a subject of careful investigation for several decades and although much more is known about specific modes of transmission for the dinoflagellate algal symbiont *Symbiodinium* similar mechanisms of transmission may be shared with other microbial symbionts. Algal symbiont transmission is thought to occur in a mainly vertical fashion in brooding corals, with larvae acquiring *Symbiodinium* from the mother polyp before release (Trench, 1983; Baker, 2003). Vertical transmission has also been observed in several spawning corals that seed their eggs with *Symbiodinium* (Apprill et al., 2009; Padilla-Gamino et al., 2012). In contrast, externally fertilized larvae generally acquire *Symbiodinium* from the environment (horizontal transmission) (Trench, 1983; Baker, 2003).

Although less is known about transmission of other non-*Symbiodinium* microbial symbionts in the holobiont, several studies document both vertical transmission (transfer of symbionts from parent to offspring) and horizontal transmission (uptake of symbionts from the environment) of Bacterial populations. Vertical transmission in *Porites astreoides* was recently demonstrated. Ectoderm-associated bacteria from the parent colony, mainly *Roseobacter* spp., were observed in both newly released, and in 4-day old planulae, with densities increasing after settlement (Sharp et al., 2012). In contrast, many coral larvae appear bacteria-free after fertilization and only acquire bacteria as planula or polyps. Apprill et al. (2009) found that 3-day old, but not newly released, planulae of *Pocillopora meandrina* contained internalized bacterial cells; with *Roseobacter* spp., as the primary constituents. Horizontal transmission was also documented in the study by Sharp et al. (2010) which followed gametes and planulae of seven broadcast spawners and found no bacterial associations until after the first polyp stage.

Recent studies point to the water column and to the mucus layer surrounding the gametes of spawning corals as media for transfer of symbionts (*Symbiodinium* and bacterial species) from parent colony to larvae after gamete release (Apprill et al., 2009; Ceh et al., 2012, 2013; Sharp et al., 2012). Both brooding and broadcast spawning corals were observed to release bacteria during spawning, comprised primarily of *Roseobacter* spp. and *Alteromonas* spp. (Ceh et al., 2013). The mucus coats of gamete bundles are associated with bacterial populations similar to those found in the parent colony leading to speculation that vertical transmission of these populations may occur either prior to spawning by seeding of the mucus coat with maternally-derived bacterial symbionts, or post-spawning through uptake of microbial associates released by the parent colony into seawater (Apprill et al., 2009; Ceh et al., 2012, 2013; Sharp et al., 2012).

Beneficial activities of the bacterial holobiont members acquired during spawning are unknown and remain an important avenue for ongoing studies. Antimicrobial activity of resident microbes may serve a protective function for coral spawn or newly hatched larvae, although antimicrobial activity associated with extracts from egg bundles themselves was found in only 1 of 11 coral species (*Montipora digitata*) from the Great Barrier reef (Marquis et al., 2005). As pointed out by Sharp and Ritchie (2012), *M. digitata* uniquely among the 11 coral species tested, seeds its egg bundles with *Symbiodinium*, leading the authors to speculate a potentially protective role for *Symbiodinium*-derived metabolites. Although a low prevalence of solvent-extractable antimicrobial activity was observed in this study, it is possible that other protective compounds such as colonization inhibitors, or antimicrobial peptides (AMP), may contribute to the protection of spawn and larvae against uncontrolled bacterial colonization, as has been suggested by studies in adult corals and in the Cnidarian, Hydra (Jung, 2009; Vidal-Dupiol et al., 2011; Franzenburg et al., 2013; Krediet et al., 2013).

### Settlement and metamorphosis

Despite a substantial number of studies investigating the potential role of microorganisms in the induction of larval settlement and metamorphosis, a thorough understanding of all the mechanisms and players involved is still lacking. In corals, metamorphosis and settlement are tightly coupled processes where a coral individual transitions from a motile planula larva to a sedentary polyp. Emerging evidence suggests that bacteria have a fundamental role in moderating metamorphosis and settlement of larvae in the marine environment and suggest that this event may be triggered by diffusible or potentially contact-mediated signals (Dobretsov and Qian, 2004; Hadfield, 2011; Dobretsov et al., 2013; Shikuma et al., 2014).

Enhanced larval settlement and metamorphosis has been observed in response to crustose coralline algal (CCA) surfaces (Morse et al., 1988) that represent a consortium of a red alga and a complex community of bacteria and archaea. Bacterial isolates from CCA surfaces (*Pseudoalteromonas* spp.) were found to form biofilms that were sufficient to increase metamorphosis rates of *Acropora willisae* and *A. millepora* by up to 50% (Negri et al., 2001). Complex bacterial biofilms formed from shallow reef waters were also sufficient to induce high levels of larval settlement and metamorphosis for *A. micophthalma* and were superior to biofilms formed in deep-water suggesting a connection between spatial variability of marine biofilm community composition and efficiency of larval settlement (Webster et al., 2004). Finally, antibiotic treatment of larval cultures was shown to be sufficient to inhibit settlement, suggesting that bacterial activity is necessary for settlement induction (Vermeij et al., 2009).

Efforts to uncover the molecular mechanism behind induction of coral metamorphosis by various *Pseudoalteromonas* strains isolated from CCA surfaces led to isolation of the inducing compound tetrabromopyrrole (TBP) (Tebben et al., 2011). TBP was sufficient for inducing metamorphosis of *A. millepora* at environmentally relevant concentrations. However, larvae induced by TBP, or by *Pseudoalteromonas* biofilms, metamorphosed without settlement (Tebben et al., 2011). In contrast, *A. millepora* larvae exposed to complex CCA both metamorphosed and settled, leading the authors to speculate that TBP production by *Pseudoalteromonas* strains has a net negative effect on coral recruitment and may have antifouling properties (Tebben et al., 2011). In contrast, a recent study of a diverse set of Caribbean corals documented that TBP extracted from *Pseudoalteromonas* isolates, as well as produced synthetically, stimulated both metamorphosis and settlement (Sneed et al., 2014). Thus, it is clear that TBP is a diffusible signal produced by coral reef *Pseudoalteromonas* strains that stimulates coral larval metamorphosis, however TBP may have divergent impacts on net larval recruitment depending on the timing of larval settlement.

Coordination of metamorphosis and settlement may involve detection of multiple types of signals produced by biofilms and CCA including both diffusible signals such as TBP and contact mediated signals located on biofilm surfaces. A contact-mediated mechanism for induction of larval settlement of *Hydroides elegans*, a polychaete worm, was recently established for a *Pseudoalteromonas* strain (*P. luteoviolacea*)—where settlement is triggered by larval contact with bacterial cell surface displays of phage-tail like proteins (Huang et al., 2012; Shikuma et al., 2014). Certain populations of bacteria may also deter coral settlement through diffusible signals, for example larvae were observed to avoid settling on tiles adjacent to benthic cyanobacteria hypothesized to produce toxic secondary metabolites (Kuffner et al., 2006). It is likely that the combination of CCA and biofilms comprised of specific bacterial population(s) produce multiple signals that together provide adequate settlement cues for coordinated metamorphosis and settlement in coral larvae. Such coordinated cues may help larvae from diverse invertebrate taxa identify sites optimal for recruitment, and may represent long standing relationships between larvae and bacterial partners in biofilms on marine surfaces (Hadfield, 2011).

### Coral colony formation

Once settlement and metamorphosis is achieved, the coral recruit (i.e., juvenile polyp) must overcome competition with other benthic organisms to grow and form a robust adult colony. Bacteria may mediate the survival of coral recruits in the proximity of adult coral colonies or other coral competitors such as macro- and turf algae (Smith et al., 2006; Marhaver et al., 2013). Enrichment of pathogenic and opportunistic bacteria in the vicinity of adult coral colonies plays a macro-scale role in reef community structure through enhanced mortality of nearby conspecific coral recruits (Marhaver et al., 2013). This effect, first described as the Janzen-Connell hypothesis for diversification of terrestrial forests and may also contribute to the high diversity of invertebrate species observed in coral reefs (Marhaver et al., 2013).

Enrichment of bacteria near primary producers such as benthic algae may also negatively impact the survival of coral recruits. Elevated mortality (up to 100%) of coral nubbins grown with algal competitors and appeared to be mediated by factors able to pass through a fine mesh (Smith et al., 2006). These negative effects were mitigated by the addition of the antibacterial compound ampicillin (Smith et al., 2006) suggesting that bacterial activities, rather than algal toxins, mediated the inhibitory effects of algal proximity on coral recruits. A similar result was obtained by Vermeij et al. (2009), who examined the post-settlement survival of *Montipora capitata* in the presence and absence of macroalgae and ampicillin where the addition of ampicillin increased recruit survivorship from around 30 to 60% (Vermeij et al., 2009). Exudation of organic carbon by primary producers may enrich for heterotrophic bacteria (Discussed further in Section How can Ecological Approaches Deepen our Understanding of the Multiple Levels of Coral–Microbial Interactions?), including pathogens and opportunists that may impact the survival of recruits. Early survivorship of coral recruits can be increased through gregarious settlement in a species-specific manner (Rivera and Goodbody-Gringley, 2014) and whether coral-to-coral allorecognition or competition during settlement is influenced by coral associated microbial populations is a question worthy of investigation.

### Sexual maturation and reproduction

Once settled, recruits grow and reach reproductive maturity. Brooding corals tend to breed year round, releasing smaller quantities of well-developed larvae, while spawning corals release gametes in large, often synchronous, spawning events a few times a year. Timing is generally thought to be mediated by the lunar cycle in most species (Babcock et al., 1986; Tanner, 1996). Over the course of spawning events the composition of bacterial assemblages associated with adult colonies remains relatively stable (Apprill and Rappe, 2011; Ceh et al., 2012) with a notable exception being an increased abundance of several microbial populations hypothesized to play a role during reproduction (Ceh et al., 2012, 2013). During spawning Ceh et al. (2012) observed increases in the proportion of α-Proteobacterial 16S rRNA gene sequences (particularly *Roseobacter* spp.) in the microbiota of three different coral species: *Acropora tenuis, Pocillopora damicornis*, and *Tubastrea faulkneri*, and subsequently documented increases of *Roseobacter* spp. and *Alteromonas* spp. in filtered seawater concurrent with release of gametes from *A. tenuis*, or larvae from *P. damicornis* (Ceh et al., 2013). The authors hypothesized that release of these microbial populations enriched the bacterioplankton with seed populations for colonization of gametes and larvae. Speculation on potential beneficial functions for these reproductive-associated bacteria include antimicrobial protection and roles in induction of larval settlement (Ceh et al., 2013) however the nature of mutualistic interaction, if any, remains unknown.

Coral spawn that does not proceed through development, decays and represents a source of nutrients and carbon to the water column and benthos that may stimulate growth of microbial populations. During a 4-day mass spawning event in Heron Island on the Great Barrier Reef the abundance of bacteria in the water column nearly doubled and increased 3-fold in the sediments (Patten et al., 2008). The impact of increased microbial activity and abundance stimulated by spawn decay on reef water quality is mediated by the physical characteristics of the reef region i.e., dilution capacity and flushing rate. In shallow bays of the Great Barrier Reef blooms of respiring bacteria, and subsequent hypoxia, in response to decaying coral spawn have been associated with mass mortalities of corals and other benthic invertebrates (Simpson et al., 1992). In contrast, in Kaneohe Bay, Hawaii, a bay with comparatively higher flushing rates, slight changes in bacterial production and microbial community composition in the water column were only observed when spawning coincided with a low tide where the dilution-capacity of the bay was at a minimum (Apprill and Rappe, 2011). Release of nutrients and bacterial populations during coral spawning may impact the early colonization of planulae by bacterial associates although this impact is likely to be influenced by physical process such as flow, mixing, and initial nutrient loads.

## How is homeostasis maintained between corals and their microbial symbionts?

**Figure 2:**
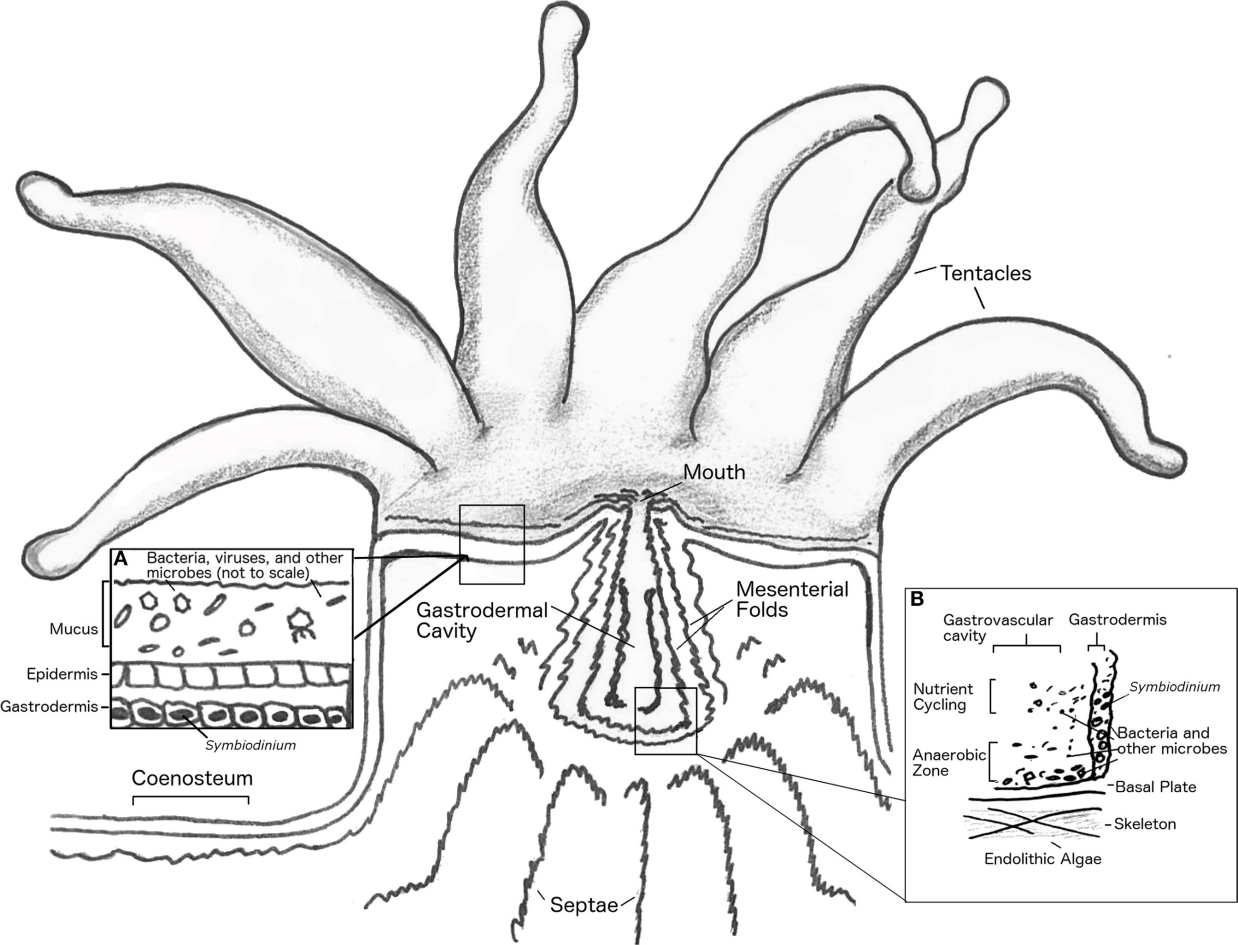
**Anatomy of coral polyp**. Basal body wall not shown. Inset **(A)**, shows close up of mucus layer, epidermis and upper gastrodermis. Inset **(B)**, shows close up of coral gastrovascular cavity.

The coral holobiont, from pre-larval to adult stages, is a complex system of diverse organisms that coexist and interact. This complex system is capable of fixing nutrients and building biomass and carbonate structures in nutrient poor waters. When one or more components of the holobiont fail to function as required for system stability, bleaching (*Symbiodinium* loss) and/or tissue-death may result and lead to progressive death of the entire coral colony. The control and regulation of activities in the holobiont to maintain emergent function is termed homeostasis. In the coral holobiont, like any other animal holobiont, stressors that threaten homeostasis may be biological, chemical, or physical, and an initial stressor may lead to cascading interactions that trigger additional responses. To assess how homeostasis is maintained among components of the coral holobiont this section will focus on how physical and chemical gradients shape the microbiota in different habitats within the coral animal, and consider how perturbation of these gradients influence holobiont function. In particular, we will examine the following habitats: (1) the coral mucus and epidermal surface, (2) intracellular and interstitial spaces, (3) the gastrovascular cavity, and (4) the skeleton (Figure 2).

### Coral mucus and epidermal surface

The tissue of the coral organism contains numerous secretory cells that are responsible for production of a continuous mucus layer that is typically a few hundred micrometers thick (Brown and Bythell, 2005; Jatkar et al., 2010; Garren and Azam, 2012). The formation and roles of coral mucus have been reviewed in detail (Brown and Bythell, 2005). Coral mucus serves multiple functions to maintain holobiont homeostasis that may vary in relative importance depending on the coral species. As a permeable barrier between the coral tissue and seawater that can be periodically shed by a mechanism functionally analogous to mucus clearing in other metazoans, the mucus layer is the first line of defense against biofouling, pathogen invasion, and shading by sedimentation (Brown and Bythell, 2005). In addition, the mucus layer may serve as a trap for nutrient-bearing particles (including bacteria) that are periodically ingested by the coral as a mode of suspension feeding (Coles and Strathman, 1973; Muscatine, 1973). Mucus layers are enriched in photosynthate and coral waste products and thus represent a resource-rich habitat that is readily colonized by marine bacteria (Brown and Bythell, 2005). Mucus layers accumulate dense assemblages of bacteria that share similarity with the bacterial composition of seawater (Bourne and Munn, 2005; Lema et al., 2012) and are also structured by interaction with members of the coral holobiont through both top-down process (i.e., removal/selection) (Ritchie, 2006; Franzenburg et al., 2013) and bottom-up processes (i.e., resource availability) (Nakajima et al., 2009) as discussed further in Section How can Ecological Approaches Deepen our Understanding of the Multiple Levels of Coral–Microbial Interactions? Coral mucus layers are complex habitats, defined by chemical, physical and biological gradients (Brown and Bythell, 2005), thus it is not surprising that spatial heterogeneity of mucus-associated bacterial community composition in adult corals can be observed over the scale of a single coral colony (Daniels et al., 2011). Indeed, over a diurnal cycle the mucus of some corals may cycle from being fully-oxygenated due to the photosynthetic metabolism of *Symbiodinium* to anaerobic at night when respiration rates exceed oxygenesis—this may be enhanced by physical compartmentalization of mucus-bearing structures on the coral colony (Carlton and Richardson, 1995; Kuhl et al., 1995; Brown and Bythell, 2005).

Although the mucus layer is densely colonized by microorganisms, microscopic examination of the coral epidermis beneath the mucus layer reveals a nearly sterile environment (Johnston and Rohwer, 2007). Surface coral cilia have been implicated in deterring the attachment of fouling organisms by generating surface currents on the order of 2 mm per second (Johnston and Rohwer, 2007; Garren et al., 2014). Although coral mucus and ciliary currents are physical deterrents to colonization of coral surfaces, it has been recently demonstrated that the coral pathogen *V. coralliilyticus* breaches this barrier through rapid bursts of chemotaxis (i.e., directional swimming relative to a gradient) and chemokinesis (i.e., acceleration in response to a chemical concentration) guided by gradients of infochemicals such as DMSP that are produced by the holobiont and may increase during periods of temperature stress (Garren et al., 2014). Exposure to *V. coralliilyticus* leads to rapid death of coral tissue (Ben-Haim et al., 2003a; Santos et al., 2011). Thus, the dynamic coral mucus layer is a critical, and traversable, barrier that is essential to maintaining a healthy balance of surface-colonizing microorganisms.

### Coral cells and interstitial spaces

In corals, the intracellular habitat of endodermal (gastrodermal) cells is dominated by *Symbiodinium*, which are housed in coral generated membrane vesicles i.e., the “symbiosome” as individuals, diads, or triads (Trench, 1983, 1993). The secondary membrane around the algal endosymbiont protects the alga from coral immune responses and creates a microenvironment within the cytoplasm of low nutrients and high pCO_2_/low pH conducive to exudation of photosynthate rather than algal biomass synthesis (Trench, 1983, 1993; Venn et al., 2009). To date, studies with detailed ultrastructural imaging of the symbiosome do not report the co-occurrence of bacteria (e.g., Venn et al., 2009; Pernice et al., 2012). Yet obtaining axenic (bacteria-free) cultures of *Symbiodinium* has proven difficult, suggesting that *Symbiodinium* associated bacteria may be essential for their sustained growth in culture (Ritchie, 2011). Despite efforts to remove microbial contamination from the culture, analysis of the *Symbiodinium* genome also revealed the presence of alphaproteobacterial sequences (Shoguchi et al., 2013). This organism matched closely to *Parvibaculum lavamentivorans*, and to sequences found in seawater samples (Schleheck et al., 2011). Whether *Symbiodinium* maintains intracellular bacteria is unknown.

The activity of *Symbiodinium* may shape several intracellular and interstitial physicochemical gradients in the coral holobiont. Reactive oxygen production is associated with the activity of coral phagocytotic immune cells (Mullen et al., 2003) and with oxygenic photosynthesis by *Symbiodinium* that generates free oxygen radicals in and around coral gastrodermal cells (Kuhl et al., 1995; Banin et al., 2003). Resulting gradients of reactive oxygen within the holobiont may structure microbial populations according to their ability to mitigate oxidative stress through enzymatic activity [e.g., superoxide dismutase (SOD) and catalase]. Virulence of *Vibrio shiloi*, a pathogen of *Symbiodinium* in the coral *Oculina patagonica* required SOD to initiate infection (Banin et al., 2003). While a mutant *V. shiloi* strain with inactivated SOD could adhere to and penetrate cells of *O. patagonica*, intracellular survival was impaired, preventing establishment of infection (Banin et al., 2003). These results suggest that high reactive oxygen concentrations proximal to *Symbiodinium* are a barrier to microbial colonization of the intracellular niche, and may play a role in mediating colonization of microorganisms elsewhere in the holobiont where reactive oxygen is produced.

A second class of physicochemical gradients may be shaped by exudation of organic molecules by intracellular *Symbiodinium*. In addition to photosynthate, which may represent a source of nutrition for associated bacteria, an increasing diversity S*ymbiodinium-*derived of chemical compounds has been recognized. For instance, *Symbiodinium* produce microsporine amino acids, which localize to the coral mucus and are hypothesized to protect the holobiont against UV radiation (Banaszak et al., 2000) and to serve as antioxidants (Yakovleva et al., 2004). *Symbiodinium* also produce DMSP, which is metabolized by a wide variety of microbial taxa (Raina et al., 2009) and as previously discussed, serves as an infochemical for chemotactic pathogens (Garren et al., 2014). How the profile of compounds released by *Symbiodinium* varies based on clade or sub-clade is not well-known but may influence the composition of the associated microbiota. Coral microbial communities have been shown to differ based on the clade of *Symbiodinium* present in juveniles of two Acroporid coral species under both stressed and non-stressed conditions (Littman et al., 2009, 2010) supporting the hypothesis that unknown factors associated with different *Symbiodinium* genotypes influence the coral microbiota composition.

Residence of bacterial populations in intracellular habitats affords close proximity to host- or *Symbiodinium*-derived ATP and nutrients, and protection from wandering immune cells and predators. In most known cases intracellular occurrence of bacteria in corals has been linked to pathogenesis (e.g., *V. shiloi, V. coralliilyticus*) (Ben-Haim et al., 1999, 2003a,b; Banin et al., 2003). However, intracellular association of non-pathogenic bacteria has been observed in two Cnidarian classes—the hydrozoans *Hydra magnipapillata* and *Hydra oligactic* and the anthozoan coral *Montastrea cavernosa* (Lesser et al., 2004; Fraune and Bosch, 2007; Chapman et al., 2010) and has been implicated in cultivation-independent surveys of holobiont microbiota composition. The genome sequence of *H. magnipapillata* revealed a near complete genome for a betaproteobacterial strain (family Comamonadaceae) with some evidence of genome-reduction associated with symbiosis (Chapman et al., 2010). In *H. oligactic*, stable associations with *Rickettsiales* were observed in both laboratory and field polyps that were harbored in secondary membranes within epithelial cells suggesting these were endosymbionts (Fraune and Bosch, 2007). In *M. cavernosa*, a nitrogen-fixing cyanobacterium was found within coral cells (Lesser et al., 2004) and subsequently shown to supplement the nitrogen demand of the host (Lesser et al., 2007). The abundance of *Symbiodinium* has been positively correlated with the abundance of nitrogen-fixing bacteria (Olson et al., 2009) which may provide a substantial nutritional benefit to all members of the coral holobiont. Association of nitrogen-fixing bacteria with corals have been reported in several other studies (e.g., Chimetto et al., 2008; Lema et al., 2012; Olson and Lesser, 2013). Nitrogen-fixing taxa include various rhizobia closely related to intracellular nitrogen-fixing symbionts of legumes, suggesting rhizobial coral associates may play a similar role (Lema et al., 2012).

Bacterial aggregates associated with coral gastrodermal tissue have been documented in early studies of both reef-building corals and anemones (reviewed in Work and Aeby, 2014). Among 21 coral genera examined, prevalence of cell-associated microbial aggregates (CAMA) was linked to coral genera; notably CAMA were absent in all samples examined from the genus *Montipora* (Work and Aeby, 2014). Based on histological staining profiles the authors speculated that some of the bacteria resident in CAMA were *Rickettsia* or *Chlamydia*—two taxonomic groups containing obligate intracellular species. CAMA are currently not known to be intracellular or extracellular based on current ultrastructural characterization. Widespread association of bacterial aggregates with cells of adult corals, whether intra- or extra-cellular, may point to functional mutualism, and further work to elucidate the species composition and activity associated with these aggregates will shed light on this interesting hypothesis.

### Gastrovascular cavity and lumen

**Figure 3:**
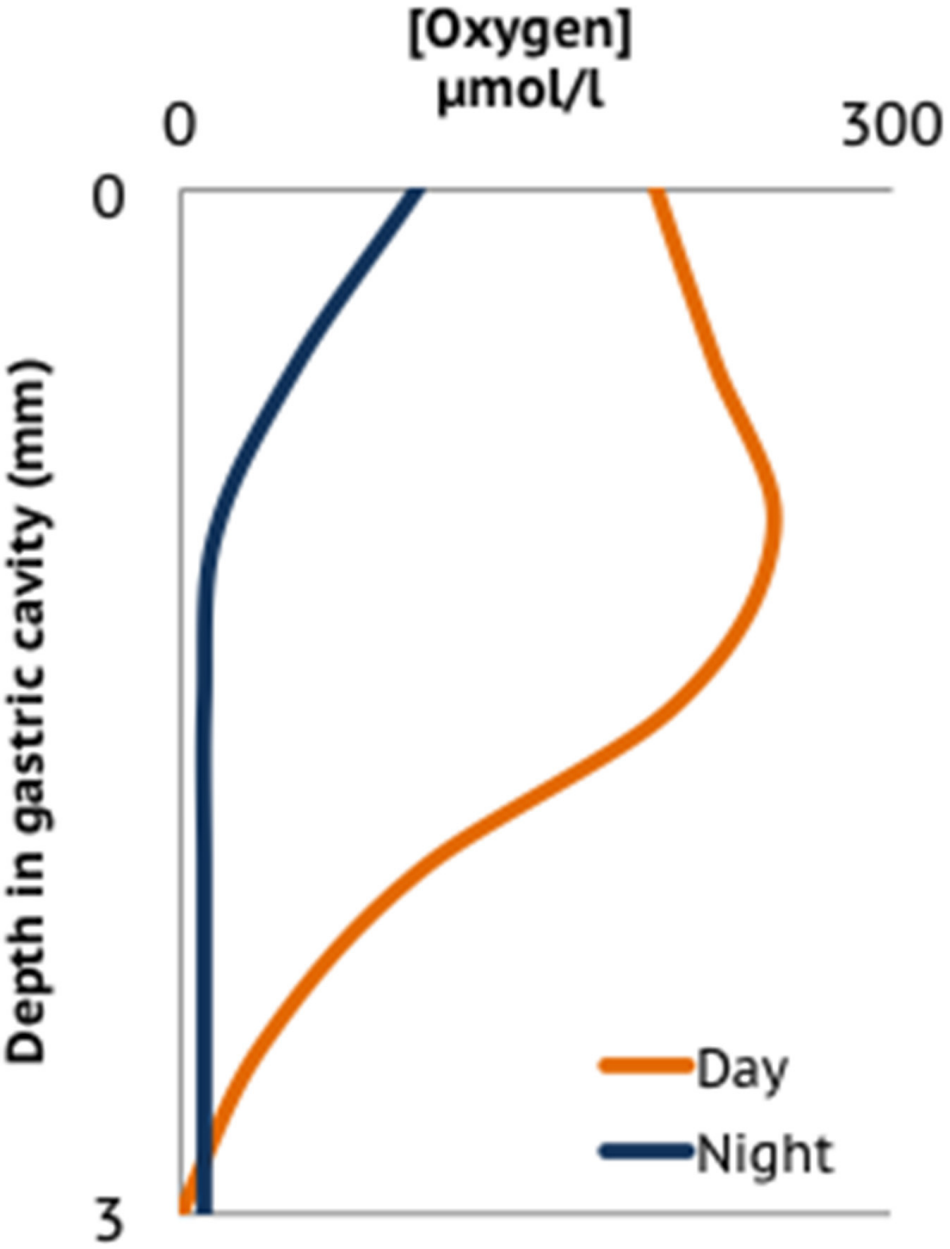
**Oxygen profile in the gastrovascular cavity of a coral polyp adapted from averaged data presented in Agostini et al. (2012)**.

The coral gastric cavity and associated lumen form an enclosed environment with properties distinct from surrounding reef waters and other coral tissues. The gastrovascular cavity is exposed to periodic influxes of both nutrients and a rich assemblage of microbial populations borne in seawater, detritus consumed via suspension feeding, and on coral prey items. Microorganisms in ingested detritus or coral prey may represent transiently associated populations and are hypothesized to contribute to the large diversity of microorganisms observed associated with corals (Fernando et al., 2014). Similar to guts of other organisms, the microorganisms in the coral gastrovascular cavity may provide their host with essential nutrients such as vitamins and amino acids, while participating in food digestion. Using fiber optic microsensors in the only study of its kind to date, Agostini et al. (2012), measured the physical properties at different depths in the gastric cavity of *G. fascicularis* and observed gradients in dissolved O_2_, pH, alkalinity, and nutrient concentrations that varied with diel oxygenic photosynthesis of associated *Symbiodinium*. Elevated levels of vitamin B_12_, phosphate, and nitrogen species were observed in the gastric cavity, where vitamin B_12_ was hypothesized to be a nutrient produced by bacteria for coral or *Symbiodinium* uptake. Dissolved oxygen decreased from the coral mouth to the bottom of the cavity, where consistently low or anoxic conditions prevailed, perhaps creating a permanently anoxic microenvironment (Agostini et al., 2012) (Figure 3) and overall, pH was lower in the cavity compared to overlying seawater. The microbial community in gastric fluids was 100-fold more concentrated than in the surrounding seawater (i.e., >10^7^ cells/ml) and the community composition included ribotypes observed in gut microbiomes of other marine and terrestrial taxa (shrimp, bees, and humans) (Agostini et al., 2012). This data suggests that the coral gastrovascular cavity may be more similar to other animal guts in terms of microbiota structure, and possibly function, than previously expected.

### Coral skeleton

The coral skeleton provides a sheltered environment, offering protection from stressors such as UV light and predation (Shashar et al., 1997). Several studies have examined microbial composition of coral skeletons, finding diverse, and distinct communities of microbial eukaryotes (Shashar et al., 1997), and bacterial communities that contain many of the same bacterial types observed in coral fragments with live tissue (Frias-Lopez et al., 2002; Sweet et al., 2011; Fernando et al., 2014). In a study of bacterial diversity of *Mussismilia* spp. corals, Fernando et al. (2014) observed that 8.9% of the bacterial 16S rRNA operational taxonomic units (OTUs) from 12 coral individuals were also observed in a bare skeleton sample. Interestingly, skeleton-associated OTUs made up a greater proportion of the bacterial richness for sequence types found in multiple coral samples, up to 77% of the richness of OTUs recovered from at least 11 of the 12 corals (Fernando et al., 2014), suggesting that skeleton-associated microbial populations are a significant source of diversity in the coral holobiont and may contain stable associates. Coral skeletons from three coral species maintained in aquaria revealed bacterial community profiles that did not cluster in a host species-specific manner, in contrast to mucus and tissue samples (Tremblay et al., 2011) raising interesting questions about the extent to which conditions in the coral skeleton are influenced by interactions with the other habitats in the coral holobiont. In 11-day old juvenile polyps of *P. astreoides* bacterial cells observed by fluorescent *in situ* hybridization are seen adjacent to tissues surrounding developing coral skeleton (Sharp et al., 2012). The reason for this localization is unclear, though the authors suggest future work should explore whether bacteria may influence deposition of the carbonate skeleton in developing corals. Indeed, the mineral nucleating properties of bacterial cell wall material is well-established and can be both induced in the laboratory, and observed in natural mineral formations (Aloisi et al., 2006).

The activity of the endolithic microbial community is elusive; far fewer studies have focused on skeletal associates than on tissue- and mucus-associated bacteria. Skeleton-associated microbes have been implicated in nitrogen fixation and as a potential source of food and nutrients during bleaching events (Shashar et al., 1994; Le Campion-Alsumard et al., 1995a; Fine and Loya, 2002). Endolithic algae can be observed macroscopically, and blooms are triggered when coral paling or bleaching reduces competition with *Symbiodinium* for light. Endolithic algae can be recognized as green bands a few millimeters beneath the coral tissue layer (Fine et al., 2006; Carilli et al., 2010). The endolith *Ostreobium* is distributed among a wide diversity of corals and as a micro-borer can negatively affect the robustness of the coral skeleton (Lukas, 1974; Gutner-Hoch and Fine, 2011). Fungi are also observed in microscopic examination of coral skeletons (Le Campion-Alsumard et al., 1995b). Coral associated fungal assemblages are diverse (Wegley et al., 2007; Amend et al., 2012), can change composition based on environmental conditions (Le Campion-Alsumard et al., 1995b; Bentis et al., 2000; Amend et al., 2012; Yarden, 2014), increase relative to other holobiont members in stressed corals (Vega Thurber et al., 2009; Littman et al., 2011), and in some cases cause coral disease (Bourne et al., 2009). While it is likely that microbial communities in the coral skeleton are distinct from those in tissue-associated habitats due to different physicochemical conditions and localized primary production by endolithic algae, the nature of interactions between endolithic microbiota and the rest of the holobiont is unclear as there are not sufficient studies with both the spatial and taxonomic resolution to distinguish microbial populations associated with live tissue from those associated with healthy coral skeletons.

## How can ecological approaches deepen our understanding of the multiple levels of coral-microbial interactions?

**Figure 4:**
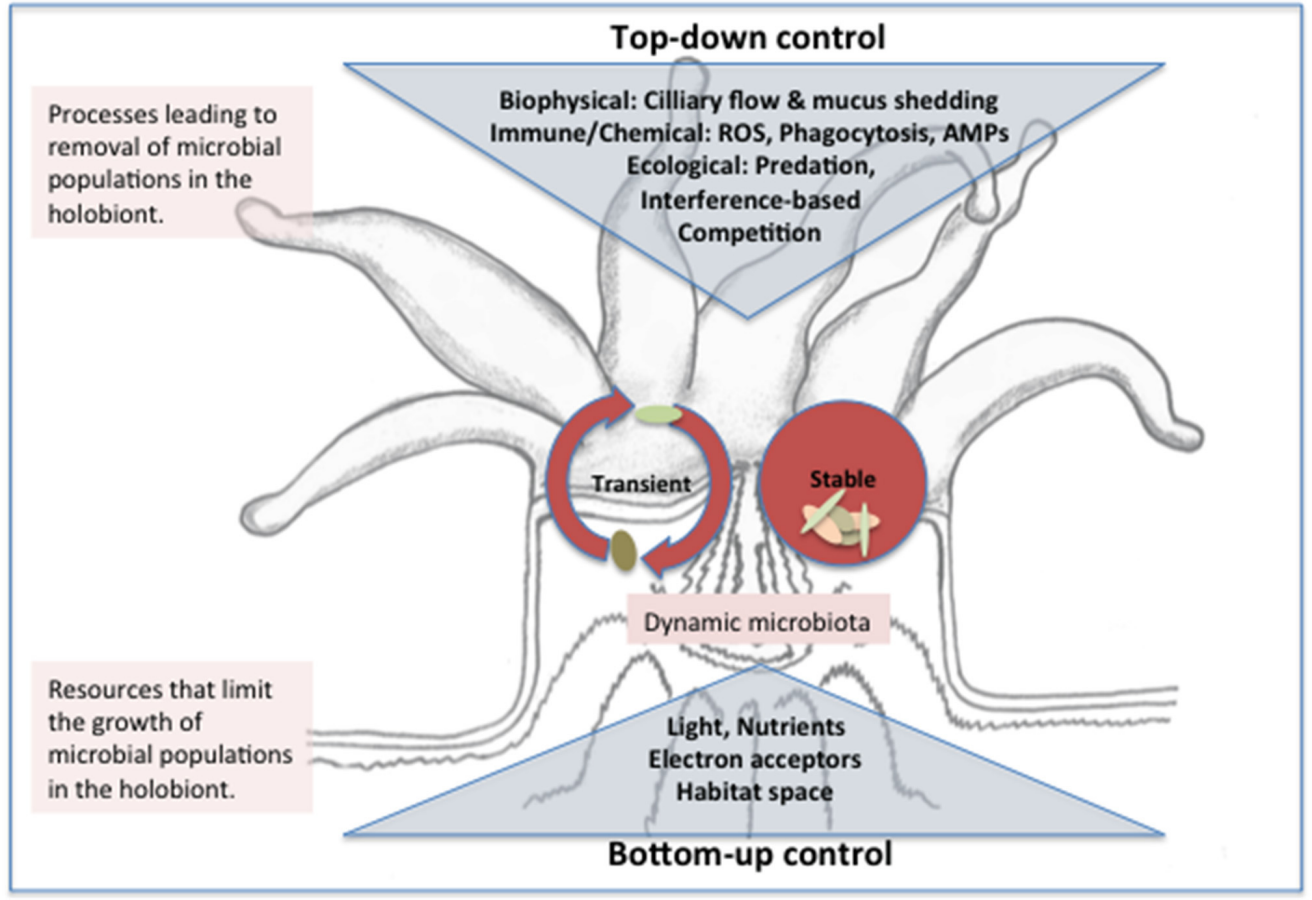
**Conceptual model of top and bottom down control of the microbiota structure in the coral holobiont**. Stable microbes may be introduced to the holobiont through horizontal or vertical transmission and persist in ecological niches within the coral polyp where growth (or immigration) rates balance removal pressures from biophysical processes and immune or ecological interactions. Transient microbes enter the holobiont from environmental sources (e.g., seawater, prey items, or suspension feeding) and removal rates exceed growth/immigration rates such that a dynamic and high diversity microbiota results. Transient and stable populations compete for resources including nutrients, light and space and the outcome of resource-based competition (bottom up control) ultimately determines population growth rate and thus ability to persist when subject to removal. Whether a population is categorized as stable or transient may depend on the timeframe considered. Abbreviations: AMP, antimicrobial peptides; ROS, reactive oxygen species.

The coral microbiota structure reflects the outcome of structuring in the distinct physicochemical micro-environments within the coral holobiont (Section How is Homeostasis Maintained between Corals and their Microbial Symbionts?) and ecological interactions. In homeostasis, microbial populations are subject to the balance of resource limitation (bottom-up control) and antagonistic interactions that lead to population extinction (top-down control) (Figure 4). Deviations from homeostasis arise when the balance of top-down and bottom-up processes is perturbed, e.g., through alteration of environmental conditions. Cascading responses may in turn lead to growth of pathogens or opportunistic microorganisms, shutting down emergent functions and destroying the scaffold of the coral holobiont with ecosystem-scale consequences.

### Bottom-up control of microbial community structure and activity through resource availability

The microbial communities of several species of corals are hypothesized to harbor stable associates of the coral host, including strains of the nitrogen-fixing cyanobacteria *Cyanothece* (Lesser et al., 2004, 2007; Olson and Lesser, 2013), alphaproteobacterial strains, such as *Roseobacter* spp., which are transferred from parent to offspring during reproduction, (Sharp et al., 2010, 2012; Ceh et al., 2013), antimicrobial members persisting in the coral mucus such as *Exiguobacterium* spp., *Photobacterium* spp., and *Halomonas* sp. (Ritchie, 2006; Krediet et al., 2013) and populations such as *Endozoicomonas* spp. that are found at high proportion across multiple types of corals (Yang et al., 2010; Morrow et al., 2012, 2014; Bayer et al., 2013a,b; Pike et al., 2013; Neave et al., 2014). Resources for maintenance metabolism coupled with favorable physical and chemical conditions are necessary for any organism to persist in a habitat. If the organism is subject to removal (e.g., by predation, or physical dilution) then biomass growth must balance removal rates for persistence. In the coral holobiont, like other ecosystems, competition for resources including light, inorganic nutrients (N, P, and Fe), electron acceptors (O_2_, SO_4_), and organic carbon mediates the structure of the populations that persist and coexist. Perturbations that relieve resource limitation have both holobiont and ecosystem scale consequences.

#### Competition for light and electron acceptors in the holobiont

Light and electron acceptors are critical resources that exert bottom-up control on interactions between coral hosts and bacteria. While the role of light as a resource for *Symbiodinium* is quite apparent, photosynthetic microbes in the holobiont such as the cyanobacteria and anoxygenic phototrophs also need access to light energy for growth (Lesser et al., 2004; Magnusson et al., 2007). Microbial phototrophs likely compete with *Symbiodinium* for light—indeed shading by the pigments in the coral polyp can be so severe that epiphytic algae residing in the skeleton beneath the coral tissue of *Porites* spp. are estimated to receive 1–4% of incident photosynthetically active radiation (PAR) (Shashar and Stambler, 1992) and values as low as 0.1% transmitted PAR have been reported for *Favia* corals (Shibata and Haxo, 1969).

As discussed previously (Section How is Homeostasis Maintained between Corals and their Microbial Symbionts?), oxygenic photosynthesis renders most of the coral interior oxic during the day, however diurnal patterns of anoxia/hypoxia in the polyp gastrovascular cavity (Agostini et al., 2012), coral surface (mucus layer) (Shashar et al., 1993; Brown and Bythell, 2005), skeleton (Kuhl et al., 2008), and in coral tissues compromised by contact with stagnant water or sediment (Kuhl et al., 1995; Weber et al., 2012), enables anaerobic forms of bacterial respiration and fermentation to occur within the holobiont. Bacterial diversity surveys of the coral holobiont have consistently recovered sequences most similar to anaerobic bacteria (Williams et al., 1987; Carlton and Richardson, 1995; Kuhl et al., 1995; Kellogg, 2004; Kline et al., 2006; Closek et al., 2014). While many aerobic heterotrophic bacteria can carry out fermentation in the absence of accessible external electron acceptors, other populations can access the relatively high concentrations of dissolved sulfate in seawater as a terminal electron acceptor for sulfate reduction, producing toxic sulfides as a by-product. Although sulfate-reducing bacteria (SRB) are detected in 16S rRNA gene surveys of presumably healthy corals (Sunagawa et al., 2010; Fernando et al., 2014), toxic sulfides generated by increased growth of SRB can contribute to coral tissue death (Carlton and Richardson, 1995; Bourne et al., 2009, 2011; Richardson et al., 2009). SRB, in particular *Desulfovibrio* spp., are involved in the progression and transmission of Black Band Disease (Frias-Lopez et al., 2004; Richardson et al., 2009; Sato et al., 2011) and possibly other coral diseases. Thus, resource limitation for oxygen may lead to destabilizing proliferation of bacterial populations that generate sulfide.

#### Limitation of holobiont microbiota by carbon and nutrients

Corals lacking *Symbiodinium* obtain nearly all of their macro- and micronutrients from predation and suspension feeding. Symbiotic corals on the other hand supplement a major proportion of their carbon demand by utilization of exuded photosynthate, while still acquiring nutrients from external sources (Trench, 1970, 1983, 1993; Muscatine and Porter, 1977). High relative abundance of microbial cells in the coral gastrovascular cavity likely participate in food digestion in a similar manner as other animal gut microbiota (Agostini et al., 2012). Dense assemblages of microorganisms are also found in coral mucus layers, and mucus has been observed to be a carbon source for bacterial growth (Nakajima et al., 2009). Since photosynthate accumulates within intracellular symbiosomes and is passed directly to the coral host, this source of carbon may not be directly available to the heterotrophic microbiota until it is metabolized and released as waste or mucus. Thus, microorganisms that reside within the coral holobiont may encounter a feast-famine dynamic of carbon and nutrient loading depending on coral feeding behavior and mucus quality.

Macro- and micronutrient availability may also limit microbiota activity in the coral holobiont. Nitrogen limitation is indicated by the success of nitrogen-fixing cyanobacteria in the coral *M. cavernosa* (Lesser et al., 2004, 2007) and the widespread distribution of the nifH gene in reef building corals—a gene encoding a nitrogenase that mediates the costly metabolic process of nitrogen fixation (Lema et al., 2012; Olson and Lesser, 2013). Additional evidence for nutrient limitation in the holobiont comes from amendment experiments and analysis of nutrient-enrichment gradients where elevated waterborne nutrients are linked to proliferation of endolithic algae in coral skeleton (Fabricius, 2005) and increased density of *Symbiodinium* in the coral host (Marubini and Davies, 1996; Tanaka et al., 2013) (Section Disruptive Effects of Carbon and Nutrient Enrichment). Upregulated expression of transporters for nutrient uptake have recently been correlated with nutrient limitation in bacterioplanktonic populations (Harke and Gobler, 2013) and future multi-“omics” work targeting the holobiont transporter profiles of microbial populations may shed light on the global dynamics of nutrient limitation across the multiple habitats of the holobiont.

**Figure 5:**
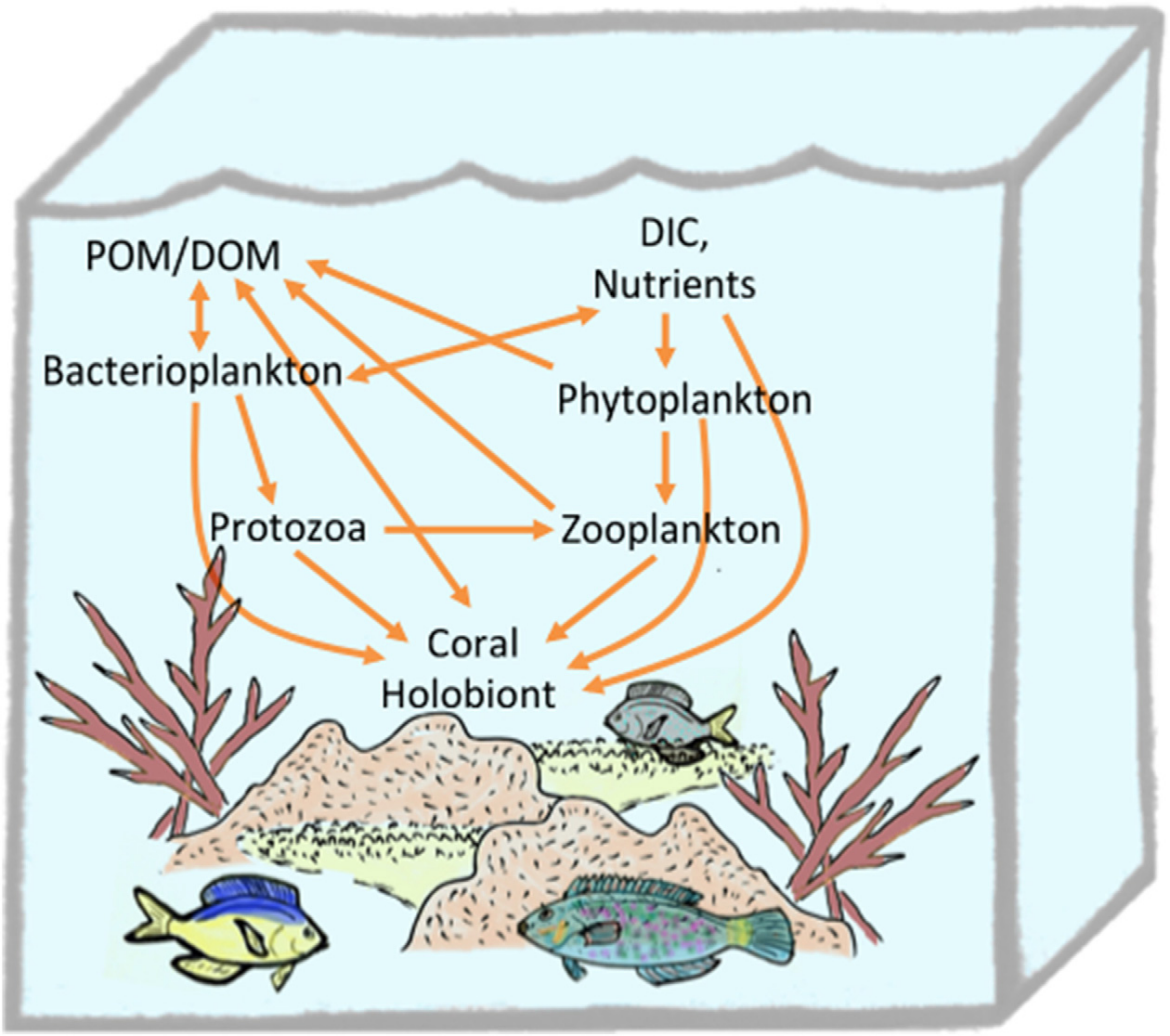
**Trophic connections of the coral holobiont in the planktonic food web**.

In addition to predation and suspension feeding, corals may obtain nutrients directly. Scleractinian corals are capable of uptaking amino acids (Drew Ferrier, 1991; Al-Moghrabi et al., 1993; Grover et al., 2008; Tremblay et al., 2012), ammonium (Burris, 1983), and dissolved and particulate organic matter from the surrounding seawater (Sorokin, 1973; Tremblay et al., 2012). Indeed, ^32^P-radiolabeling experiments with bacteria or inorganic phosphate demonstrate that six coral species assimilate phosphate directly from seawater, or as labeled bacterioplankton via suspension feeding, with higher efficiency for the latter (Sorokin, 1973). The reverse was true for carbon uptake where ^14^C-radiolabelled low molecular weight dissolved organic matter (DOM) was taken up more efficiently that the carbon-equivalent of bacterial cells (Sorokin, 1973). Microbes are well-established as agents that enable the transfer of DOM to higher trophic levels through the microbial loop (Azam et al., 1983; Azam and Cho, 1987). Assimilation of DOM into the coral holobiont may also be converted to animal biomass and mucus that is accessible to higher trophic levels (Wild et al., 2004; Naumann et al., 2010). Recently uptake of DOM and conversion to detritus by the sponge holobiont has been shown to play a major role in recycling nutrients in reef ecosystems via a cycle termed the “sponge loop” (de Goeij et al., 2013). Although photosynthetic activity by *Symbiodinium* in the coral holobiont places corals as net exporters of DOM into reef ecosystems (Wild et al., 2004), uptake of some forms of DOM by the coral holobiont contributes to efficient recycling of fixed carbon and nutrients on reef ecosystems that allows dense assemblages to thrive in nutrient poor regions (e.g., a “coral loop”) (Figure 5).

#### Disruptive effects of carbon and nutrient enrichment

Within the holobiont, and at the ecosystem scale, nutrient-limitation (oligotrophy) appears to be critical for the healthy function of most reefs dominated by coral. Perturbation of the reef environment by exposure to nutrients (N, P, and Fe) or dissolved organic carbon (DOC) has been linked to changes in microbial community structure and adverse physiological consequences for corals (Table 1). Nutrient enrichment is recognized as one of the strongest drivers of coastal habitat degradation and has been associated with increased frequencies of coral disease and bleaching (Vega Thurber et al., 2014). Studies of nutrient enrichment in experiments conducted at the local reef scale (Ward and Harrison, 2000; Koop et al., 2001; Bell et al., 2007; Vega Thurber et al., 2014), under controlled aquarium conditions (Muscatine et al., 1989; Marubini and Davies, 1996; Tanaka et al., 2013) or observations over *in situ* gradients (Loya et al., 2004) provide insights into the impacts of N and/or P addition on holobiont physiology (reviewed in Fabricius, 2005). Stimulated growth of *Symbiodinium* accompanies nutrient additions, which can lead to enhanced calcification and rates of skeletal elongation (Muscatine et al., 1989; Marubini and Davies, 1996) but also may increase the risk of bleaching after UV or thermal stress induced production of reactive oxygen by *Symbiodinium* photosystems (Lesser, 1996; Vega Thurber et al., 2014). Nutrient addition stimulates growth of endolithic algae that act as microborers and elevate rates of skeletal bioerosion, resulting in a more fragile coral skeleton (Koop et al., 2001). Other impacts linked to nutrient enrichment include reduced rates of embryogenesis and reduced overall reproductive success (Ward and Harrison, 2000; Koop et al., 2001; Loya et al., 2004; Bell et al., 2007). Nutrient amendments were associated with increased overall growth rates for *Symbiodinium*-bearing (but not aposymbiotic) acroporid coral juveniles, promoting recruitment success during competition with benthic cyanobacteria (Tanaka et al., 2013) illustrating complex outcomes depending on the duration of nutrient enrichment and coral life stage.

**Table 1:** **Effects of physicochemical shifts on the coral holobiont**.

**Stressor Direction**	**Response (and representative references)**
Organic carbon	• Growth of heterotrophic bacteria and increased abundance (Kline et al., 2006; Nelson et al., 2013)
↑	• Reduced dissolved oxygen (Simpson et al., 1992)
	• Enrichment of virulent bacteria or virulence genes (Vega Thurber et al., 2009; Nelson et al., 2013)
Nutrients (N, P or Fe)	• N/P/Fe: Increased algal growth and activity in benthos and plankton and DOM exudation (Bell et al., 2007; Kelly et al., 2012)
↑	• N/P/Fe: Enrichment of virulent bacteria or virulence genes (Vega Thurber et al., 2009; Kelly et al., 2012)
	• N/P: Increased proportion of viral sequences (Vega Thurber et al., 2008)
	• N/P: Decreased larval production (Ward and Harrison, 2000; Koop et al., 2001; Loya et al., 2004)
	• P: increased rates of skeletal extension, contributing to decreased skeletal density (Koop et al., 2001)
	• P: increased rates of bioerosion by microborers (Koop et al., 2001; Carreiro-Silva et al., 2012)
Dissolved oxygen	• Survival of dark-incubated *Montipora peltiformis* in anoxic water for up to 96 h (Mass et al., 2010).
↓	• Enhanced mortality of *Montipora peltiformis* in anoxic water with other stressors (low pH, elevated sulfide, contact with organic rich sediment)(Mass et al., 2010).
	• Nighttime anoxia at tissue/water interface of healthy corals (Shashar et al., 1993), in the gastrovascular cavity (Agostini et al., 2012), and in coral tissue consumed by black band disease (Carlton and Richardson, 1995).
Sedimentation	• Stimulated microbial activity from organic carbon and nutrients leading to reduced pH, elevated sulfide and coral tissue death (Mass et al., 2010)
↑	• Reduced mass transfer across coral surface (oxygen, nutrients, waste) (Rogers, 1990)
	• Shading and decreased photosynthesis (Rogers, 1990)
	• Increased mucus production and sloughing, along with nitrogen uptake from sediment sources (Mills and Sebens, 2004)
Light/UV	• Elevated rates of oxygenic photosynthesis (Shashar et al., 1993; Mass et al., 2010)
↑	• Incident light above photoacclimation thresholds, or associated with thermal stress, associated with formation of reactive oxygen and free radicals (Downs et al., 2002), photosystem and host tissue/DNA damage (Lesser and Farrell, 2004)
↓	• Low light: Reduced coral growth rates for species reliant on photosynthetic *Symbiodinium* for nutrition (Muscatine, 1973)
Temperature	• *Symbiodinium* loss (bleaching) (Glynn, 1991, 1993; Brown, 1997; Brandt and McManus, 2009)
↑	• Increased abundance and virulence of pathogens (Ben-Haim et al., 1999, 2003b; Banin et al., 2000; Cervino et al., 2004)
	• Increased abundance of viral sequences (Vega Thurber et al., 2008, 2009; Littman et al., 2011)
	• Decreased larval recruitment to CCA (Webster et al., 2011)
pH/pCO_2_	• Experimental pH gradient (pH 7.3 and 8.2): increased % pathogens (Meron et al., 2011)
↓ ↑	• Natural pH/pCO_2_ gradient pH 7.3–8.1: no change in % pathogens (Meron et al., 2012)
	• Natural pCO_2_ gradient: microbiota shift where proportions of *Endozoicomonas* spp. decrease while *Halomonas* spp. increase (Morrow et al., 2014).
	• Low pH: Induction of viruses (Vega Thurber et al., 2008)
	• Low pH: Decreased larval recruitment to CCA (Webster et al., 2013)

Addition of DOC, directly or indirectly via nutrient-stimulated primary production, leads to high heterotrophic bacterial abundance and degradation of reef quality (Simpson et al., 1992; Kline et al., 2006; Kelly et al., 2012; McDole et al., 2012). DOC inputs may stimulate heterotrophic microbial activity and growth on reefs in a compound-dependent manner (Nelson et al., 2013). In a recent study comparing carbon exudates of corals and their algal competitors, the composition of coral-derived carbon exudates was shown to match the profile of neutral sugars in the seawater and to exert little influence on microbial community structure after seawater amendment. In contrast, exudates from reef algae were enriched in the labile neutral sugars rhamnose, galactose, fucose, and mannose+xylose (Nelson et al., 2013) and these exudates, when added to seawater, promoted growth of a subset of bacteria enriched in virulence factors (Nelson et al., 2013). Blooms of heterotrophic respiring bacteria after DOC amendment may also have indirect effects on coral physiology, such as by depleting dissolved oxygen concentrations which may render coral tissue anoxic. Whether the negative outcomes of DOC enrichment on reef health are mediated by growth of pathogens that cause disease or by impairment of coral defenses through DOC-triggered stress (e.g., local anoxia) is unclear and indeed may be mediated by both.

### Top-down control of coral holobiont structure in a dynamic environment

Top-down forcing of holobiont structure through immune interactions, predation, and interference-based competition allows certain microbes to grow/persist in the holobiont while others are eliminated. Like all animal tissues, corals represent rich stores of nutrients kept out of bounds from potential microbial consumers by tissue barriers and immune functions. The epithelium and immune system of healthy corals are critical lines of defense to prevent indiscriminant microbial digestion of viable tissues and to allow stable coexistence of coral tissue and a microbiota. In addition, emerging research indicates that other components of the holobiont (i.e., the microbiota and perhaps *Symbiodinium*) contribute to this stable coexistence through a complex chemical ecology of antimicrobial compounds and signals that modulate microbial activity. Environmental stresses that disrupt coordinated activities of the coral holobiont pose a great risk to coral homeostasis by weakening top-down control of microbiota structure and enabling growth of opportunistic microbes that proliferate at the expense of the coral host.

#### Immune interactions

Emerging knowledge regarding immunity in corals is reviewed in Mullen et al. (2003), Augustin and Bosch (2010), Palmer and Traylor-Knowles (2012). Coral immunity includes inducible components such as migrating phagocytotic cells that destroy invading microorganisms, lectins that promote aggregation of microbial cells for efficient phagocytosis, encapsulation via prophenoloxidase (PPO)-catalyzed melanization to inactivate and contain invading pathogens, and systemic functions such as expression of chitinase and 1,2 beta-glucanase enzymes that weaken microbial cell walls (Mullen et al., 2003; Augustin and Bosch, 2010; Palmer and Traylor-Knowles, 2012). Predicted mechanisms for sensing microorganisms are emerging from coral genome sequence analysis (Shinzato et al., 2012) but have not reached the stage of functional validation. Mechanistic studies for immune defenses in *Hydra* spp. that are conserved in other metazoans shed light on potential mechanisms in corals (Augustin and Bosch, 2010; Augustin et al., 2010; Hamada et al., 2013). Such mechanisms include sensing of microbial cells in interstitial and extracellular spaces via by cell membrane-bound toll-like receptors (TLR). Microbial invasion into intracellular spaces can be sensed by enzymes detecting foreign nucleic acids (nucleotide binding and oligomerization domain like receptors) (Augustin and Bosch, 2010; Augustin et al., 2010; Hamada et al., 2013). In both cases, sensors direct a signal cascade resulting in enhanced production of antimicrobial peptides and serine protease inhibitors (Augustin et al., 2009; Jung, 2009). In *Hydra*, species-specific microbial communities and regulation of the microbiome over development is shaped by the innate immune system (e.g., TLR signaling) and maternally provisioned antimicrobial peptides (AMP) (Fraune et al., 2009; Franzenburg et al., 2013).

Although targeted studies in corals are few, production of AMPs play profound roles in mediating host-microbe interactions in other invertebrate symbioses (Zasloff, 2002; Ryu et al., 2008). Production of host-derived AMPs is emerging as a trait found among multiple cnidarian taxa. In 2006, aurelin, an AMP from the Scyphozoan *Aurelia aurita* was described revealing a novel protein structure with no homology to previously identified AMPs (Ovchinnikova et al., 2006). Subsequent work in *Hydra* revealed Hydramycin (Jung, 2009) followed by identification of multiple species-specific AMPs responsible for intra-generic differences in microbiota structure (Franzenburg et al., 2013). In 2011, damicornin, the first AMP isolated from corals was described (Vidal-Dupiol et al., 2011) and was shown to be constitutively expressed in ectodermal cells of *P. damicornis* and released in response to surface immune damage. Interestingly, damicornin gene expression was repressed in colonies challenged with *V. coralliilyticus* (Vidal-Dupiol et al., 2011), suggesting AMP repression is a potential virulence mechanism of the pathogens. Findings that *P. damicornis* extracts induced in response to mechanical stress have potent activity against the coral pathogen *V. coralliilyticus* (Geffen and Rosenberg, 2005), imply the presence of yet unknown additional inducible antimicrobial factors. A second coral-derived AMP was reported from the Brazilian octocoral *Phyllogorgia dilatata* (de Lima et al., 2013). The prevalence, diversity and significance of AMPs in coral development are only beginning to be explored.

#### Antagonistic interactions: interference based competition, predation and phage infection

While coral gene expression may lead to production of antibiotic compounds and AMPs, other members of the holobiont provide additional top-down control on holobiont structure through interference-based competition, predation and phage infection. Antimicrobial compounds associated with coral mucus and produced by resident bacteria inhibit growth of certain populations of bacteria, including those noted as opportunistic pathogens (Ritchie, 2006; Rypien et al., 2010). Symbiotic bacteria can also act to inhibit growth of other bacterial strains, often invaders that are detrimental to coral health, a relationship described as the coral probiotic hypothesis by Reshef et al. (2006). For instance, certain types of alphaproteobacteria, gammaproteobacteria, bacilli, *Pseudoalteromonas* sp., and *Pseudovibrio* sp. were found to significantly inhibit growth of *V. coralliilyticus* (Rypien et al., 2010; Vizcaino et al., 2010). Alphaproteobacteria and gammaproteobacteria were antagonist against *V. shiloi* and two members of *Pseudoalteromonas* spp. were able to inhibit *V. shiloi* growth (Rypien et al., 2010). In a study employing transposon mutagenesis and assessment of mutant fitness in the *Aiptasia* model, growth on mucus of the white pox pathogen, *Serratia marcescens*, required the enzyme N-acetyl-glucosaminidase, the activity of which could be inhibited by native coral microbiota, through a small molecule isolated from a microbial member of the coral mucus, *Exiguobacterium* sp. (Krediet et al., 2013). This study illustrates that modulation of microbial activity through interference-based competition can impact the fitness of certain microbes in the holobiont and exert control of microbial community structure, in this case through elimination of a coral pathogen.

The role of non-coral predators in the holobiont is not well-constrained although there is evidence that viruses and protozoa contribute to the mortality of coral and *Symbiodinium* cells. Ciliates have been observed infecting coral tissue leading to tissue death (Antonius and Lipscomb, 2000; Croquer et al., 2006) and preying on intracellular *Symbiodinium* leading to Brown Band Syndrome in the Great Barrier Reef (Bourne et al., 2008) and White Syndrome in Hawaiian *Montipora* corals (Work et al., 2012). Whether Ciliates are exclusively associated with coral disease or also interact with the healthy holobiont is not well-established. Other eukaryotic grazers such as Thraustochytrid protists may prey on dense assemblages of bacteria and assemble at the mucus layer (Raghukumar, 1991; Harel et al., 2008). Viruses that infect coral and *Symbiodinium* have been observed in the holobiont (Wegley et al., 2007; Marhaver et al., 2008; Patten et al., 2008; Correa et al., 2013) and may mediate the health of corals and exacerbate stressful conditions. Herpes-like viral sequences were observed in viral metagenomes from *Diploria strigosa* (Marhaver et al., 2008) and later analysis of *Porites compressa* viral metagenomes from stress and control treatments revealed a higher proportion of Herpes-like sequences under stressed conditions (i.e., low pH, elevated temperature, and nutrient enrichment) (Vega Thurber et al., 2008). Genes for Herpes-like viruses were found in other cnidarian genomes suggesting that coral viral infections are common (Vega Thurber et al., 2008).

Bacteriophage sequences have been observed in all coral viral metagenomes (Wegley et al., 2007; Marhaver et al., 2008; Correa et al., 2013) and the diversity of bacteriophage-like sequences associated with corals parallels and perhaps contributes to the diversity of the microbiota (reviewed in Vega Thurber and Correa, 2011). The presence of phage in the mucus layer of corals has been proposed as a mechanism of defense against invading microorganisms (Barr et al., 2013). Binding between phage capsid proteins [immunoglobin (Ig)-like protein domains] and glycoproteins from mucus suggest that bacteriophage are readily incorporated into mucus layers (Barr et al., 2013). The widespread distribution of Ig-like proteins in viral metagenomes from cnidarian (and human) mucus suggests that metazoan mucosal surfaces and phage coevolved to maintain phage adherence. This work presents the fascinating possibility that bacteriophage from the environment are easily incorporated into the coral mucus layer and thus, these may act as an additional defense mechanism to control holobiont microbial community structure through phage infection and lysis (Barr et al., 2013).

### Deviations from homeostasis

Variation in physicochemical conditions can have distinct impacts on the coral holobiont community. Among healthy corals the bacterial community composition has been found to vary seasonally (Koren and Rosenberg, 2006) suggesting that shifting ecological drivers (bottom up and top down control) as well as shifting composition of the source assemblages from which immigration occurs leads to a dynamic microbiota that is compatible with a state of homeostasis in the holobiont. In contrast, numerous studies document shifts in coral microbiota following perturbation of physicochemical conditions that may lead to the destabilization and ultimately death of the holobiont (Table 1). Little is known about factors that determine whether the holobiont can maintain homeostasis during, or recover from, physicochemical perturbations. A general trend in coral perturbation studies is that opportunistic pathogens including vibrios and alteromonads increase following exposure to a stressor (Vega Thurber et al., 2009; Littman et al., 2011; Meron et al., 2011) suggesting that activities of virulent microbes may accelerate the impact of a stressor, and contribute to the breakdown of holobiont functioning due to changes in host response or due to pathogenicity (Rosenberg et al., 2007; Bourne et al., 2009).

## Conclusions

Scleractinian corals have survived dramatic environmental changes during their evolutionary history including changes in sea level, pH, and temperature—however current anthropogenically-induced changes are occurring at rates unprecedented in the geological record (Glynn, 1991, 1993; Hoegh-Guldberg, 1999; Pandolfi et al., 2003; Hoegh-Guldberg et al., 2007; Mora, 2008). Whether extant corals will survive under current regimes of rapid change will depend upon their ability to adapt to new conditions on anthropogenic timescales. It is likely that coral-associated microbiota will be linked to the capacity of corals to ultimately adapt to new environmental conditions. In plant systems, it has become increasingly clear that crop improvement through microbiome optimization is preferable to plant trait selection (Coleman-Derr and Tringe, 2014). In a similar fashion coral-associated microbes may mediate the resilience of corals to stresses such as resistance to coral-specific pathogens (Reshef et al., 2006; Rosenberg et al., 2007) and perhaps to other stressors.

Our understanding of coral physiology and interaction as a holobiont is now enhanced by the expanded scientific focus on the diverse members of the coral microbiota. Access to whole genome data from the major players in a single holobiont, in particular, organisms that may be vertically transmitted from parent colony to offspring, will enable researchers to develop new, readily testable hypotheses regarding metabolic complementation, genome expansion/reduction, coevolutionary patterns, and rates of HGT across all genomes in a holobiont. Such information will yield insight into the adaptations that have enabled coral holobionts to be so successful in nutrient poor environments.

In order to motivate future work to refine our mechanistic understanding of microorganisms as coral partners through evolution, development, and in ecological interactions within the holobiont we have selected eight questions to highlight based on recent literature (Table 2). Continued research is essential to promote a detailed understanding of the mechanisms shaping the coral holobiont on ecological and evolutionary timescales as pressures emerge for reefs to acclimate and adapt to conditions of global change.

**Table 2:** **Questions for future research**.

• What are the mechanisms of nutritional and defensive mutualisms between holobiont members?
This is a fundamental question to understand the ecology and physiology of the holobiont. The answers may be of biomedical interest as novel antimicrobial compounds or mechanisms that disrupt pathogen colonization or virulence without bactericidal or bacteriostatic activity (e.g., Krediet et al., 2013) may be involved.
• What is the diversity of vertically-transmitted microorganisms in coral reproduction?
• Do these populations represent obligate or facultative mutualisms?
Novel cultivation-based approaches and genome sequencing or single-cell genomics to recover populations that resist cultivation are necessary. Such improvements will enable testing of hypotheses regarding coevolution and codiversification between corals and their microbiota.
• What is the role of *Symbiodinium* in shaping microbiota acquisition in gametes, larvae, and adult coral colonies?
• Do algae produce chemical signals that mediate allelopathic recognition or modulate colonization of the microbiota?
• How do chemical signals produced by CCA biofilms promote settlement and/or metamorphosis of coral larvae and how is receipt of these signals coordinated to optimize recruitment?
• What microbial taxa and activities are associated with coral cell-associated microbial aggregates (CAMAs) and what factors mediate their distribution in the coral polyp?
• Do the enclosed microorganisms interact with the coral tissue as mutualists, parasites, or commensals?
• Since several studies have documented lower levels of microbial colonization in *Montipora* spp. compared to other corals (Marquis et al., 2005; Work and Aeby, 2014), to what extent is antimicrobial activity specific to the coral species or their associated *Symbiodinium*?
• What role does the endolithic microbial community play in the homeostasis of the coral holobiont?
A potentially transformative avenue for inquiry is exploring the relationship between bacterial activity and skeleton formation in juvenile corals as recently suggested (Sharp et al., 2012).
• What are the molecular mechanisms of the host/holobiont stress response that allow proliferation of pathogen-like bacteria (e.g., *Vibrio* spp.*, Alteromonas* spp.)?
Emerging coral holobiont transcriptomics studies will shed new light on how the coral holobiont responds to stresses and exists as a robust and resilient system when in a healthy state.

### Conflict of interest statement

The authors declare that the research was conducted in the absence of any commercial or financial relationships that could be construed as a potential conflict of interest.
